# Beyond change scores: Employing an improved statistical approach to analyze the impact of entry fitness on physical performance during British Army basic training in men and women

**DOI:** 10.1111/sms.14610

**Published:** 2024-03-27

**Authors:** Tessa D. Maroni, Andrew G. Siddall, Carla A. Rue, Sarah C. Needham‐Beck, Faye S. Walker, Julie P. Greeves, Sophie L. Wardle, Anne Fieldhouse, Stephen D. Myers, Sam D. Blacker

**Affiliations:** ^1^ Occupational Performance Research Group University of Chichester Chichester UK; ^2^ Army Health and Performance Research Army Headquarters Andover Hampshire UK

**Keywords:** cardiorespiratory fitness, fitness testing, military, physical training, strength

## Abstract

The aim was to use a robust statistical approach to examine whether physical fitness at entry influences performance changes between men and women undertaking British Army basic training (BT). Performance of 2 km run, seated medicine ball throw (MBT) and isometric mid‐thigh pull (MTP) were assessed at entry and completion of Standard Entry (SE), Junior Entry‐Short (JE‐Short), and Junior Entry‐Long (JE‐Long) training for 2350 (272 women) recruits. Performance change was analyzed with entry performance as a covariate (ANCOVA), with an additional interaction term allowing different slopes for courses and genders (*p* < 0.05). Overall, BT courses saw average improvements in 2 km run performance (SE: −6.8% [−0.62 min], JE‐Short: −4.6% [−0.43 min], JE‐Long: −7.7% [−0.70 min]; all *p* < 0.001) and MBT (1.0–8.8% [0.04–0.34 m]; all *p* < 0.05) and MTP (4.5–26.9% [6.5–28.8 kg]; all *p* < 0.001). Regression models indicate an expected form of “regression to the mean” whereby test performance change was negatively associated with entry fitness in each course (those with low baseline fitness exhibit larger training improvements; all interaction effects: *p* < 0.001, $\eta_{p}^{2}$ > 0.006), particularly for women. However, when matched for entry fitness, men displayed considerable improvements in all tests, relative to women. Training courses were effective in developing recruit physical fitness, whereby the level of improvement is, in large part, dependent on entry fitness. Factors including age, physical maturity, course length, and physical training, could also contribute to the variability in training response between genders and should be considered when analyzing and/or developing physical fitness in these cohorts for future success of military job‐task performance.

## 1 INTRODUCTION

Soldiers are required to have high levels of fitness to perform physically demanding, role‐related, military tasks, such as prolonged load carriage and moving equipment.^1^ Developing cardiorespiratory endurance and muscular strength, and power through progressive physical training (PT) programmes during basic training (BT) is essential to ensure soldiers can effectively perform their duties. Increased physical fitness also reduces musculoskeletal injury risk during training and subsequent military service.^2^, ^3^ Given the focus on the development of cardiorespiratory and muscular endurance in traditional military training, improvements in these fitness components have been observed through routine testing across British Army BT (12–14 weeks)^4^, ^5^, ^6^, ^7^ and the BT of other nations (courses ranging 8–16 weeks).^8^, ^9^, ^10^, ^11^, ^12^, ^13^, ^14^, ^15^ Power and high‐intensity interval training has increasingly been incorporated into military training and testing as knowledge of training approaches have advanced alongside the continually changing requirements of military roles.^16^, ^17^

During BT, recruits with a range of physical fitness levels conduct training in groups; resulting in individuals working at different relative intensities to achieve the same task. The difference in training stimulus may contribute to the greater improvements in physical fitness which are often experienced in those who enter training with the lowest baseline fitness levels.^14^, ^18^ In contrast, recruits presenting high initial fitness may experience less of a stimulus, resulting in minimal improvements or even decreased physical fitness (detraining) by the end of training.^5^, ^8^, ^11^ Theoretically, this relationship between initial fitness level and subsequent training responses during BT is a form of “regression to the mean”, where extreme values from within‐subject variation on one measurement stabilize towards the sample mean on subsequent measurements.^19^ The “mean” in this case would be the desired level of fitness for a generic soldier and would likely lie marginally above the minimum physical employment standard (PES). Therefore, individualized training may provide a more positive exercise stimulus for adaptation in those commencing training with high fitness levels.^8^ However, despite knowledge of these principles present in military training, appropriate statistical analysis has rarely been employed in previous research describing changes in physical performance during BT, by not adequately considering an individual's physical performance at the start of training.

Further, in common research questions comparing between groups which are non‐randomly assigned (i.e., men and women, or across different training establishments), additional analytical considerations are required to generate unbiased statistics,^20^, ^21^ but are rarely accounted for. Women typically enter BT less physically fit than their male counterparts, but have shown absolute greater fitness improvements (almost twice the rate of men) by the end of training.^10^, ^13^, ^22^ However, a large gap in physical fitness between men and women still exists after BT, which may increase during further training and/or carryover into service. This was seen in Finnish recruits, where fitness test improvements were larger in men compared with women after 6–12 months of service, despite men commencing training with higher overall fitness levels.^23^ Currently, women account for 10.9% of the British Army^24^ and, have recently been permitted to serve in all job‐roles irrespective of the associated physical demand.^25^ Although male and female British Army recruits complete the same training programmes, women experience greater cardiovascular strain and increased injury risk during BT, which has been associated with their lower average body mass and aerobic fitness upon entry, relative to men.^4^, ^26^, ^27^ Therefore, it is imperative that the appropriate fitness capacities are developed and tested to assess training development, with the aim of enhancing the physical readiness of all soldiers.

In 2019, the British Army implemented new gender‐agnostic PES for applicants and recruits, which assess cardiorespiratory and strength parameters both upon entry (Role Fitness Test (Entry); RFT [E]) and upon completion of BT (Role Fitness Test [Basic Training]; RFT [BT]). These gym‐based predictor tests consist of a seated medicine ball throw (MBT), an isometric mid‐thigh pull (MTP) and a 2 km run, which were selected due to their collective capability to predict an applicant's ability to meet the PES for in‐service personnel (Role Fitness Test (Soldier); RFT [S]) after training. The British Army BT follows a Common Military Syllabus adapted for Standard Entry (SE) recruits (>17.5 years old, 14‐week course) as well as Junior Entry (JE) recruits (16–17.5 years old) over long (49‐weeks; JE‐Long) or short (23‐weeks; JE‐Short) courses (JE training duration is dependent on the job role recruits will perform in‐service). However, the performance changes of men and women to these specific tests, and whether training adaptations differ across recruit training courses is unknown. Understanding the extent of these differences may benefit how, or whether, training approaches could be adapted, and improve expectation and preparedness for those entering training. Taking into consideration analytical limitations of previous work and changes to fitness testing, the overarching aim of this study was to use a more considered statistical approach for determining if initial fitness influences the subsequent changes in recruits' MBT, MTP, and 2 km run performance across British Army SE, JE‐Short, and JE‐Long BT courses, and specifically, whether performance changes differ between men and women. Therefore, this paper first explores the absolute changes in fitness test performances across the courses and then presents the results when using initial fitness at entry as a covariate. This approach is repeated with a comparison between men and women, ending with an in‐depth presentation of the variance in responses for each fitness test.

## 2 METHODS

### 2.1 Participants

The individual best‐effort performance on the MBT, MTP, and 2 km run were recorded for 2350 recruits (2078 men and 272 women) at the start and end of the three British Army BT courses (SE, JE‐Long and JE‐Short) from April 2019 (inception of the tests) to February 2020. Two sources of data were used: (1) Data gathered by the researchers (272 recruits total; 242 men and 30 women; mean ± SD: age: 17.7 ± 3.3 years; body mass: 71.0 ± 10.3 kg; height: 174.4 ± 7.5 cm) and (2) Data gathered through routine Army testing conducted by Physical Training Instructors (PTIs) and, recorded on the British Army Fitness Information System Software (FISS) (2078 total; 1836 men and 242 women; physical characteristics not available). All data were anonymized once performance scores on the tests at the start and end of training had been linked. All personnel were thoroughly briefed, and participants provided written informed consent for the researchers, while the British Army provided consent to using the anonymized FISS data. Ethical approval was granted by the Ministry of Defence Research Ethics Committee (#993/MoDREC/19).

### 2.2 Experimental approach and procedures

British Army BT involves a wide variety of demanding PT including running, strength and circuit training, swimming, agility, and battle/combat PT, that progressively increase in load over the duration of training. Although the exact details of the training courses are not available for publication, PT sessions are completed multiple times per week (see Table 1), with all recruits completing the same programmes within course, in combination with military drill, adventurous training, and military skills training activity (e.g., fieldcraft, range firing, navigation, combat maneuvers, prolonged field exercise). These non‐PT activities are scheduled throughout each course, with varying degrees of intensity and duration, and recruits are often expected to transit between locations on training sites on foot, encompassing considerable additional physical activity to structured PT.

**TABLE 1:** Overview of the physical training syllabus completed by men and women during British Army standard entry (SE) and junior entry (JE) basic training courses during the study period. Data are counts.

Physical training	SE (14 weeks)	JE‐long (49 weeks)	JE‐short (23 weeks)
Aerobic development	10	11	4
Strength and conditioning	28	36	17
Swimming	10	12	5
Battle/Combat PT	8	6	3
Load carriage	28	10	7
Testing	15	3	3
Total number of sessions	99	78	39

*Note*: NB: Session duration: SE = 40 min, JE = 80 min.

Recruits completed the fitness tests at the start (RFT [E]) and end of training (RFT [BT]), in the following order: (1) MBT, (2) MTP, and (3) 2 km run, wearing PT kit and trainers. The test protocols and techniques were standardized according to the British Army Fitness test procedure policy.^28^ The MBT required recruits to complete a seated maximum distance throw of a 4 kg medicine ball, for one practice and two best‐effort attempts (distance recorded to the nearest 10 cm). For the isometric MTP, recruits stood on a weighing scale housed within a specially manufactured (for the British Army), standardized rig, with a bar fixed at mid‐thigh level, where participants took hold of the bar with an overhand grip and straight arms in a quarter‐squat position. On command, recruits exerted a maximal isometric force by pulling the bar upwards as hard as possible, maintaining the pull for ~5 s. Peak absolute force (kg) was recorded during two best‐effort attempts, which were separated by 30–60 s recovery. The MBT and MTP are commonly used in military populations as simple and reliable tests for evaluating upper and lower body strength,^29^ with the best attempt score taken for both the MBT and MTP. Finally, following an 800 m warm up, recruits completed a 2 km run (to assess cardiorespiratory capacity) on a flat track at an individual best‐effort pace (time recorded to the nearest second).

### 2.3 Statistical analyses

All analysis was conducted using commercial software: SPSS Statistics for Windows (Version 25.0. Armonk, NY: IBM Corp), R^30^ and RStudio.^31^ Performance data of each course were checked for normality using the Shapiro–Wilk test and the change in predictor test performance over training (start to end) were assessed using paired sample *t*‐tests. We initially used an analysis of covariance (ANCOVA) to determine the overall impact of the covariate (entry performance) on performance change across each course. Use of an analysis of covariance (ANCOVA), is generally preferable to analyzing change scores which ignore the impact of baseline scores on outcome.^32^ Our study is observational and a classification design, where attendance at different training establishments represented pre‐classified groups, and there was no control group. Theoretically then, post‐BT performance scores were influenced by a combination of entry fitness, (non‐randomized) group and training, as well as unknown factors which we acknowledge could introduce untested/uncontrolled biases (e.g., nutrition, injury status). In this type of design (when groups are classified prior to the study (not randomized) and/or differ non‐randomly at baseline), between‐group difference estimates from ANCOVA can be biased or even contradictory. Therefore, we added an interaction term to the traditional ANCOVA model to compare the relationship between entry fitness and change in fitness (a) between courses and, subsequently, (b) between men and women within each course. As this interaction model allows independent slopes, the homogeneity of regression slopes assumption for traditional ANCOVA is redundant. Estimated marginal means were generated using the {emmeans} R package^33^ for the 5th, 50th, and 95th percentile of entry scores in each test to demonstrate how between‐group effects differ along the regression line. All other assumptions for performing ANCOVA were tested: comparison groups were independent; approximate homogeneity of variances (via Levene's test), normality of residuals (via Shapiro–Wilk) and homoscedasticity (by visual inspection). As it would be impossible to assess all assumption violations in an observational human field trial, *p*‐values for associations are interpreted as a measure of compatibility between the observed data and the entire underlying null test model (i.e., null hypothesis of no association and collective statistical assumptions used to compute the *p*‐value).^34^ Data are presented as mean ± standard deviation (SD) or as adjusted (estimated marginal) mean and 95% “compatibility” (confidence) intervals (CI), where appropriate. For general linear model outputs, standardized effect sizes (Partial eta‐squared ($\eta_{p}^{2}$)) are presented with exact *p*‐values (unless *p* < 0.001) to allow reader interpretation.

## 3 RESULTS

In absolute terms, there was group average improvement on all performance measures but, in many cases, the standard deviation of responses was larger than the change seen over training for 2 km run (SE: −0.62 ± 0.77 min [−6.8%]; JE‐Short: −0.43 ± 0.84 min [−4.6%]; JE‐Long: −0.70 ± 0.92 min [−7.7%]; all *p* < 0.001), MBT (SE: 0.04 ± 0.58 m [1.0%]; JE‐Short: 0.31 ± 0.42 m [8.4%]; JE‐Long: 0.34 ± 0.44 m [8.8%]; *p* < 0.001) and MTP (SE: 6.5 ± 30.5 kg [4.5%]; JE‐Short: 16.6 ± 20.5 kg [13.7%]; JE‐Long: 28.8 ± 33.6 kg [26.9%]; all *p* < 0.001).

In all courses, entry performance, as a covariate, was associated with subsequent change in 2 km run (*F*_(1,2346)_ = 1512.583, $\eta_{p}^{2}$=0.392 *p* < 0.001), MBT (*F*_(1,2346)_ = 737.133, $\eta_{p}^{2}$=0.239, *p* < 0.001) and MTP (*F*_(1,2346)_ = 1169.857, $\eta_{p}^{2}$=0.333 *p* < 0.001) performance. In a regression between performance change and entry fitness, a negative slope suggests some regression to the mean either via within‐subject variability or such that participants with poorer entry fitness improve more during training than those with higher fitness (or a combination of both). We observed this with the traditional ANCOVA model, with negative gradients on baseline‐adjusted slopes for change in 2 km run (slope and 95% CI: −0.463 [−0.486 to −0.439]), MBT (−0.410 [−0.439 to −0.380]) and MTP (−0.518 [−0.548 to −0.488]). Crudely, these slopes indicate estimated change in fitness from baseline improves by ~0.4–0.5 units (i.e., minutes, metres, kilograms) with every whole unit decrease in fitness at entry. In this traditional ANCOVA model, the slopes for each course are restricted to being parallel, which does not reflect that the courses themselves are separate population blocks with different characteristics.

**FIGURE 1:**
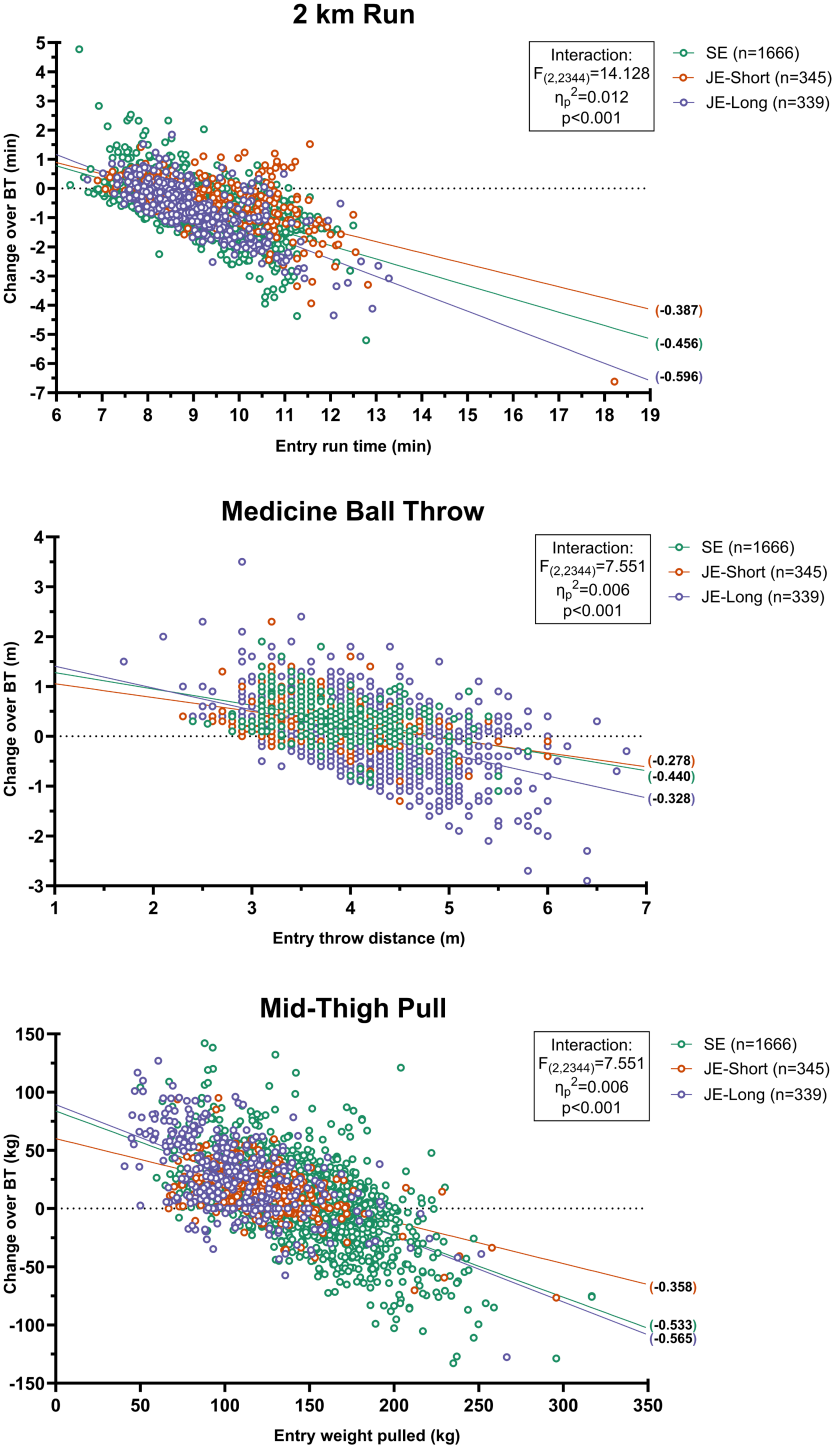
Scatterplots of individual performance change across basic training courses (SE, JE‐Short and JE‐Long) for the 2 km run (top), medicine ball throw (middle) and mid‐thigh pull (bottom) in relation to entry performance score. Regression lines represent the changes in test performance across each course on the basis of entry fitness. Details on individual panels display gradients for slopes for the fitted regression model and the associated Entry × Course interaction effects.

When an interaction model was used, slopes appeared to differ by course for all three tests (Figure 1) indicating that performance at entry may be associated with expected average change in fitness differently in each course, but with high inter‐individual variability. Estimated marginal means from this model for the cohort 5th, 50th, and 95th percentile of entry performance demonstrates the differing impact of entry fitness on change within the same test (Table 2). For example, in SE for those with a run time of 11:00 min the compatibility interval for the estimated mean change is an improvement of between 1.41–1.59 min (1:24–1:35 min:s), in contrast to those with a run time of 7.6 min (7:36 min:s), whose compatibility interval surrounds zero (−0.01–0.14 min). The courses with the steepest slopes, and therefore the largest putative association between entry fitness and subsequent change, was JE‐Long for 2 km run (−0.596 [−0.655 to −0.536]) and SE for MBT (−0.440 [−0.474 to −0.407]). For MTP, the slope for JE‐Short appeared shallower (−0.358 [−0.445 to −0.270]) than the other two, suggesting improvement (or lack of) was less related to entry MTP performance in the shorter course duration.

**TABLE 2:** General linear model characteristics for baseline fitness‐adjusted change in fitness for each course within test. Estimated marginal means are produced from the slopes and intercepts with 95% compatibility intervals (CI) for the 5th, 50th, and 95th percentile entry scores for each test.

	Model					Estimates (interaction model for entry score against change)			Estimated marginal mean change (CI) by entry score percentile		
Test	Effect	df	*F*	*p*‐value	$\eta_{p}^{2}$	Course	Intercept (CI)	Slope (CI)	5th percentile	50th percentile	95th percentile
Run (min)	Intercept	1	845.493	<0.0001	0.265				**11.0 min**	**9.0 min**	**7.6 min**
	Entry fitness	1	1152.556	<0.0001	0.330	SE	3.51 (2.91, 4.12)	−0.456 (−0.485, −0.427)	−1.50 (−1.59, −1.41)	−0.59 (−0.63, −0.55)	0.06 (−0.01, 0.14)
	Course	2	9.697	<0.0001	0.008	JE‐Short	3.21 (2.48, 3.93)	−0.387 (−0.437, −0.336)	−1.05 (−1.19, −0.90)	−0.27 (−0.37, −0.18)	0.28 (0.12, 0.44)
	Entry × *Course	2	14.128	<0.0001	0.012	JE‐Long	4.73 (4.19, 5.28)	−0.596 (−0.655, −0.536)	−1.82 (−2.01, −1.64)	−0.63 (−0.73, −0.54)	0.22 (0.06, 0.38)
	Residuals	2344									
	Intercept	1	357.861	<0.0001	0.132				**3.1 m**	**4.0 m**	**5.1 m**
	Entry fitness	1	251.956	<0.0001	0.097	SE	1.85 (1.48, 2.22)	−0.440 (−0.474, −0.407)	0.48 (0.43, 0.54)	0.09 (0.06, 0.12)	−0.40 (−0.45, −0.34)
MBT (m)	Course	2	4.390	0.013	0.004	JE‐Short	1.33 (0.86, 1.81)	−0.278 (−0.366, −0.189)	0.47 (0.37, 0.57)	0.22 (0.14, 0.30)	−0.08 (−0.27, 0.11)
	Entry × *Course	2	7.551	0.001	0.006	JE‐long	1.61 (1.26, 1.95)	−0.328 (−0.416, −0.240)	0.59 (0.47, 0.71)	0.29 (0.22, 0.37)	−0.07 (−0.24, 0.10)
	Residuals	2344									
	Intercept	1	967.018	<0.0001	0.292				**80 kg**	**136 kg**	**194 kg**
	Entry fitness	1	567.658	<0.0001	0.195	SE	83.7 (73.9, 93.5)	−0.533 (−0.568, −0.498)	41.1 (37.5, 44.7)	11.3 (9.5, 13.0)	−19.8 (−22.7, −16.8)
MTP (kg)	Course	2	9.404	<0.0001	0.008	JE‐Short	60.1 (46.3, 73.8)	−0.358 (−0.445, −0.270)	31.4 (25.2, 37.7)	11.4 (7.4, 15.5)	−9.4 (−19.2, 0.3)
	Entry × Course	2	7.551	0.001	0.006	JE‐Long	89.3 (81.0, 97.7)	−0.565 (−0.639, −0.491)	44.1 (39.5, 48.7)	12.5 (7.7, 17.2)	−20.4 (−30.3, −10.6)
	Residuals	2344									

*Note*: Percentiles for run are presented in the intuitive direction to match medicine ball throw (MBT) and mid‐thigh pull (MTP) such that better (faster) performance represent the higher percentile scores. A negative change in run is therefore an improvement in performance in contrast to the other two tests.

Abbreviations: JE, Junior Entry; MBT, medicine ball throw; MTP, mid‐thigh pull; SE, Standard Entry.

**FIGURE 2:**
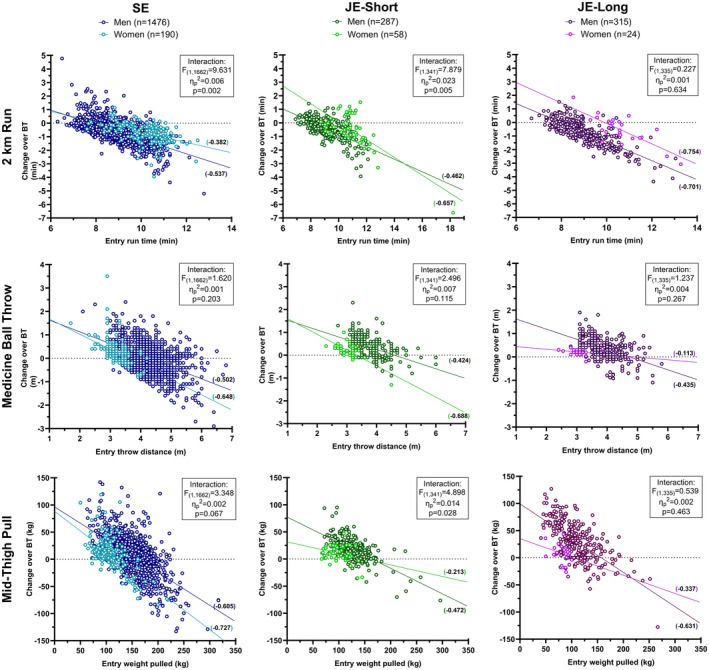
Scatterplots of individual performance change for men and women over basic training courses (SE, JE‐short and JE‐long) on the 2 km run (top row), medicine ball throw (middle row) and mid‐thigh pull (bottom row) in relation to entry performance. Regression lines represent the changes in test performance on the basis of entry fitness, separately for men and women within each course. Details on individual figures display gradients for slopes and the associated Entry × Gender interaction effects.

Unadjusted test performances by gender across courses are shown in Table 3. In most cases, women had lower average fitness at entry and broadly similar average improvement with the exception of MBT in SE (men: 0.2% [−0.5% to 0.9%] vs. women: 9.2% [7.1%–11.3%]) and MTP in JE‐Long (men: 27.8% [24.4%–31.3%] vs. women: 10.1% [1.5%–18.7%]). When permitting independent slopes (and intercepts), the negative gradients on all slopes (Table 3) are consistent with the impact of entry performance on performance change but the magnitude varies between men and women on the majority of tests and courses (Figure 2; Interaction effect descriptions noted on figure panels; see Table 4 for full model descriptions). During SE, the impact of entry fitness on performance change was greater in women for the MBT and MTP, but greater for men in the 2 km run. The gradients suggest the association with physical performance at the start of BT and/or regression to the mean for JE‐Short appears similar across all tests for men, but more severe in women for MBT and 2 km run, and less severe in MTP. A similar regression to the mean between genders seemed to occur during JE‐Long for the 2 km run, with slopes that were approximately parallel. Although individuals with lower physical performance at the start of training tended to improve more than those with higher physical performance to different degrees across gender, the shallow regression slope for MBT in women during JE‐Long suggests it was least influenced by entry ability.

**TABLE 3:** Unadjusted average changes (Δ) in fitness test performance across Basic Training (start: RFT (E) to end: (RFT (BT)) courses by gender, and estimates (slope and intercept) from the interaction model between entry fitness and change and estimated change at the 5th, 50th, and 95th percentile of fitness per course. Data are mean ± SD or estimated marginal mean (95% CI).

				Unadjusted raw scores			Estimates (interaction model for entry score against change)		Estimated marginal mean change (CI) by entry score percentile		
Test	Course	Gender	n	RFT(E)	RFT(BT)	Mean Δ	Intercept (CI)	Slope (CI)	5th Percentile	50th percentile	95th Percentile
Run (min)									**10.8 min**	**9.0 min**	**7.5 min**
	SE	M	1476	8.92 ± 0.95	8.31 ± 0.73	−0.61 ± 0.78	4.18 (3.19, 5.17)	−0.537 (−0.635, −0.439)	−1.63 (−1.72, −1.54)	−0.64 (−0.68, −0.6)	0.15 (0.07, 0.22)
		W	190	10.18 ± 0.91	9.45 ± 0.82	−0.73 ± 0.69	3.16 (2.21, 4.11)	−0.382 (−0.474, −0.289)	−0.97 (−1.11, −0.84)	−0.27 (−0.46, −0.08)	0.29 (−0.06, 0.64)
									**11.6 min**	**9.1 min**	**7.7 min**
	JE‐Short	M	287	9.09 ± 1.06	8.68 ± 0.79	−0.41 ± 0.74	3.79 (2.35, 5.24)	−0.462 (−0.599, −0.325)	−1.58 (−1.83, −1.33)	−0.43 (−0.52, −0.33)	0.22 (0.06, 0.38)
		W	58	10.93 ± 1.35	10.41 ± 0.99	−0.53 ± 1.25	6.66 (5.35, 7.97)	−0.657 (−0.776, −0.539)	−0.98 (−1.22, −0.73)	0.66 (0.3, 1.02)	1.58 (1.02, 2.14)
									**11.1 min**	**8.9 min**	**7.6 min**
	JE‐Long	M	315	9.01 ± 1.04	8.29 ± 0.62	−0.72 ± 0.91	5.60 (3.31, 7.89)	−0.701 (−0.918, −0.484)	−2.19 (−2.37, −2.00)	−0.66 (−0.74, −0.58)	0.27 (0.13, 0.41)
		W	24	10.60 ± 1.07	10.07 ± 0.71	−0.52 ± 1.05	7.46 (5.24, 9.69)	−0.754 (−0.963, −0.545)	−0.91 (−1.24, −0.58)	0.73 (0.18, 1.29)	1.74 (0.84, 2.63)
MBT (m)									**3.1 m**	**4.1 m**	**5.2 m**
	SE	M	1476	4.23 ± 0.60	4.24 ± 0.58	0.01 ± 0.58	2.13 (1.41, 2.85)	−0.502 (−0.726, −0.277)	0.58 (0.50, 0.65)	0.07 (0.04, 0.11)	−0.48 (−0.54, −0.41)
		W	190	3.14 ± 0.32	3.43 ± 0.42	0.29 ± 0.45	2.32 (1.63, 3.02)	−0.648 (−0.868, −0.427)	0.32 (0.22, 0.41)	−0.33 (−0.63, −0.03)	−1.04 (−1.66, −0.42)
									**2.9 m**	**3.6 m**	**4.5 m**
	JE‐short	M	287	3.81 ± 0.51	4.15 ± 0.48	0.34 ± 0.44	1.95 (0.93, 2.98)	−0.424 (−0.752, −0.097)	0.72 (0.61, 0.84)	0.42 (0.36, 0.49)	0.04 (−0.05, 0.14)
		W	58	3.05 ± 0.30	3.24 ± 0.23	0.19 ± 0.29	2.29 (1.31, 3.26)	−0.688 (−1.005, −0.370)	0.29 (0.15, 0.43)	−0.19 (−0.45, 0.08)	−0.81 (−1.44, −0.18)
									**3.1 m**	**3.9 m**	**4.9 m**
	JE‐long	M	315	3.93 ± 0.53	4.28 ± 0.49	0.35 ± 0.45	2.06 (0.32, 3.79)	−0.435 (−1.004, 0.135)	0.71 (0.60, 0.81)	0.36 (0.3, 0.42)	−0.08 (−0.20, 0.04)
		W	24	3.01 ± 0.27	3.22 ± 0.27	0.21 ± 0.13	0.55 (−1.15, 2.26)	−0.113 (−0.677, 0.451)	0.20 (−0.01, 0.42)	0.11 (−0.59, 0.82)	0.00 (−1.46, 1.46)
MTP (kg)									**90 kg**	**145 kg**	**200 kg**
	SE	M	1476	149.8 ± 31.1	155.8 ± 26.9	6.0 ± 30.4	96.6 (81.4, 111.7)	−0.605 (−0.736, −0.473)	42.2 (38.6, 45.7)	8.9 (7.2, 10.6)	−24.4 (−27.5, −21.2)
		W	190	107.8 ± 27.3	117.8 ± 24.7	10.0 ± 30.8	88.4 (74.5, 102.3)	−0.727 (−0.852, −0.602)	23.0 (17.5, 28.4)	−17 (−24.8, −9.3)	−57 (−73.2, −40.8)
									**82 kg**	**118 kg**	**166 kg**
	JE‐short	M	287	126.3 ± 28.6	144.0 ± 22.4	17.8 ± 21.4	77.4 (53.8, 100.9)	−0.472 (−0.702, −0.242)	38.7 (34, 43.4)	21.5 (18.9, 24.1)	−1.2 (−5.5, 3.2)
		W	58	97.7 ± 19.1	108.5 ± 20.0	10.8 ± 13.9	31.6 (9.6, 53.6)	−0.213 (−0.433, 0.008)	14.2 (6.8, 21.5)	6.4 (−1.9, 14.7)	−3.8 (−25.0, 17.4)
									**58 kg**	**103 kg**	**165 kg**
	JE‐long	M	315	109.2 ± 35.3	139.5 ± 28.9	30.3 ± 34.1	99.2 (34.8, 163.7)	−0.631 (−1.42, 0.158)	62.4 (55.7, 69.0)	34.6 (30.7, 38.5)	−5.1 (−12.2, 2.0)
		W	24	80.3 ± 13.3	88.4 ± 18.1	8.1 ± 16.4	35.2 (−28.7, 99.0)	−0.337 (−1.121, 0.448)	15.5 (−11.5, 42.4)	0.7 (−26.5, 27.8)	−20.5 (−111.6, 70.5)

*Note*: The interaction model here is a general linear model of change in test performance against gender, with entry performance as a covariate, allowing gender slopes to differ. The general linear model characteristics for baseline fitness‐adjusted change in test fitness for gender within each course are presented in Table 4. Percentiles for run are presented in the intuitive direction to match medicine ball throw (MBT) and mid‐thigh pull (MTP) such that better (faster) performance represent the higher percentile scores. A negative change in run is therefore an improvement in performance in contrast to the other two tests.

Abbreviations: JE, junior entry; M, men; MBT, medicine ball throw; MT, mid‐thigh pull; RFT(BT), Role Fitness Test (basic training); RFT(E), Role Fitness Test (Entry); SE, Standard Entry; W, women.

**TABLE 4:** General linear model characteristics for baseline fitness‐adjusted change in test fitness for gender within each course.

Test	Course	Effect	df	*F*	*p*‐value	$\eta_{p}^{2}$
Run	SE	Intercept	1	211.095	<0.0001	0.113
		Entry Fitness	1	337.099	<0.0001	0.169
		Gender	1	4.109	0.0428	0.002
		Entry fitness × Gender	1	9.631	0.0019	0.006
		Residuals	1662			
	JE‐Short	Intercept	1	201.606	<0.0001	0.372
		Entry fitness	1	259.281	<0.0001	0.432
		Gender	1	15.161	0.0001	0.043
		Entry fitness × Gender	1	7.879	0.0053	0.023
		Residuals	341			
	JE‐Long	Intercept	1	126.313	<0.0001	0.274
		Entry fitness	1	174.100	<0.0001	0.342
		Gender	1	2.568	0.1100	0.008
		Entry fitness × gender	1	0.227	0.6344	0.001
		Residuals	335			
MBT	SE	Intercept	1	147.5532	<0.0001	0.082
		Entry fitness	1	0.274145	0.6006	0.000
		Gender	1	100.7417	<0.0001	0.057
		Entry fitness × Gender	1	1.619589	0.2033	0.001
		Residuals	1662			
	JE‐Short	Intercept	1	66.351	<0.0001	0.163
		Entry fitness	1	44.537	<0.0001	0.116
		Gender	1	0.413	0.5208	0.001
		Entry fitness × gender	1	2.496	0.1151	0.007
		Residuals	341			
	JE‐Long	Intercept	1	8.761	0.0033	0.025
		Entry fitness	1	3.573	0.0596	0.011
		Gender	1	2.917	0.0886	0.009
		Entry fitness × Gender	1	1.237	0.2668	0.004
		Residuals	335			
MTP	SE	Intercept	1	573.226	<0.0001	0.256
		Entry fitness	1	396.483	<0.0001	0.193
		Gender	1	1.120	0.2900	0.001
		Entry fitness × Gender	1	3.348	0.0674	0.002
		Residuals	1662			
	JE‐Short	Intercept	1	82.821	<0.0001	0.195
		Entry fitness	1	34.219	<0.0001	0.091
		Gender	1	14.601	0.0002	0.041
		Entry fitness × Gender	1	4.898	0.0275	0.014
		Residuals	341			
	JE‐Long	Intercept	1	16.804	<0.0001	0.048
		Entry fitness	1	5.827	0.0163	0.017
		Gender	1	3.822	0.0514	0.011
		Entry fitness × Gender	1	0.539	0.4634	0.002
		Residuals	335			

Further, taking into account the potential noise generated from interaction effects, in comparison to simple main effects, although *p*‐values show low compatibility, the effect sizes for interactions are relatively smaller ($\eta_{p}^{2}$ range: 0.001–0.023) than the main impact of entry fitness alone ($\eta_{p}^{2}$ range: 0.011–0.432; Table 4). Despite some of the largest interaction effect sizes being reported in the JE courses, such direct comparisons were not drawn due to considerably lower sample numbers for women than men, and therefore we may not have sufficient data to infer a legitimate difference in slope between the two JE courses. With this possible imprecision in mind, the combination of higher mean change in men and differing slopes suggest that the models tended to show lower estimated improvement or, worse, detraining in women at the 5th, 50th, and 95th percentile cohort entry scores (Table 3), and exceptionally wide compatibility intervals for some estimates.

**FIGURE 3:**
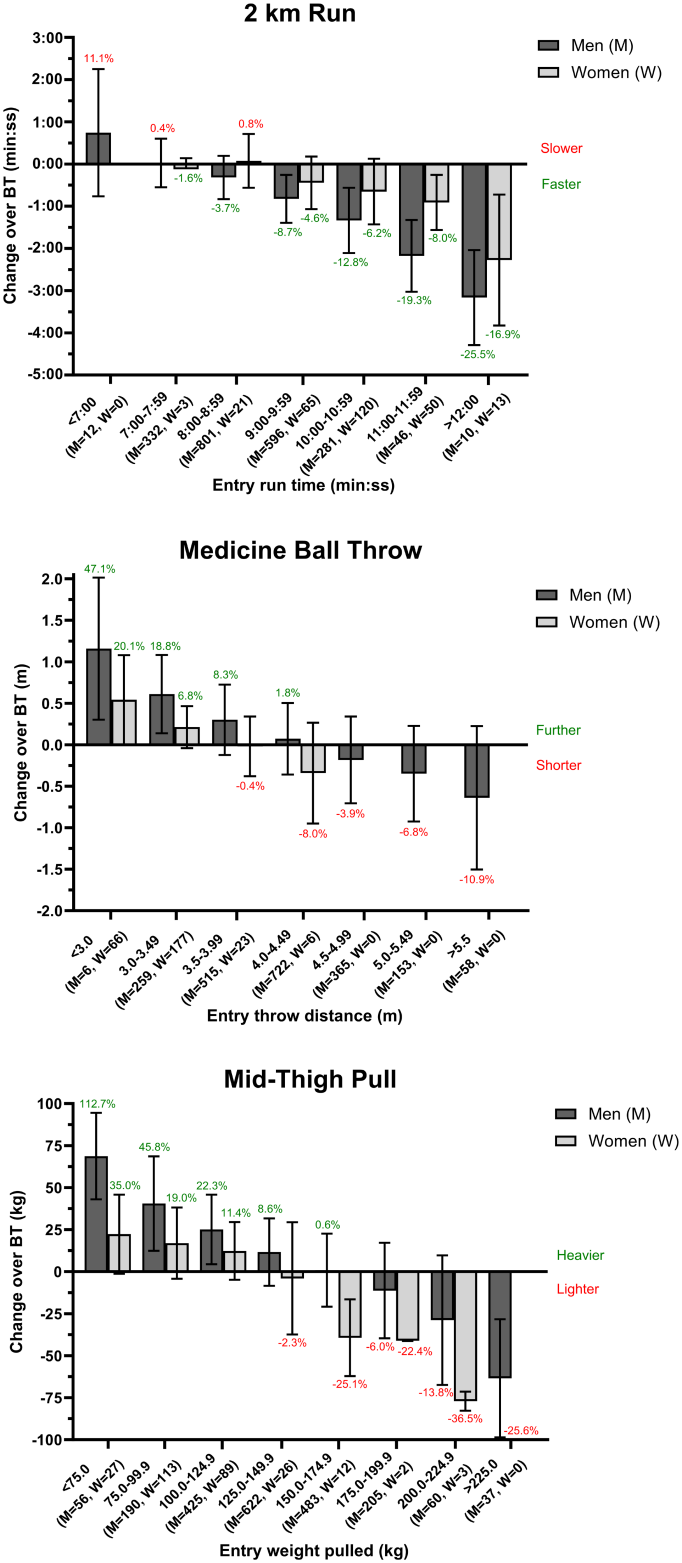
Mean ± SD (and %) changes in 2 km run (top), medicine ball throw (middle) and mid‐thigh pull (bottom) performance of combined standard entry (SE) and junior entry (JE) recruits (men and women) over basic training, when stratified by entry fitness.

To explore the relationship between gender and changes in physical performance further, we combined course data (SE, JE‐Short, and JE‐Long) and pooled men and women into groups based on physical performance at the start of training (Figure 3). The balanced pattern of improvement in individuals with lower fitness and decrement in individuals with higher fitness was evident, particularly with the wider spread of entry performances in men. Overall, men displayed considerable improvements in 2 km run, MBT and MTP, relative to women, when commencing training at a lower fitness. Whereas the decrements/minimal improvements over training for those with higher fitness was particularly pronounced in women for the MTP.

## 4 DISCUSSION

In this study we quantified the changes in physical performance of men and women during British Army SE, JE‐Long and JE‐Short BT courses, with a particular focus on using statistical approaches to quantify the impact of entry fitness. On average, 2 km run, MBT and MTP performance improved over BT, with the greatest improvements seen in recruits after a longer period of training (JE‐Long). Further, an expected form of regression to the mean was evident, where change in fitness was negatively associated with fitness at entry (those with low baseline fitness exhibit larger improvements). While performance improved for both men and women, the association between entry performance and subsequent change tended to be larger in women than men; resulting in smaller estimated gains and greater detraining in women with low and high physical fitness at the start of training, respectively. The mechanisms to explain the variability in training adaptations between men and women across the courses (particularly in large recruit cohorts) may be a combination of factors including entry fitness and training status, course length and composition, age and physical maturity, component of fitness tested, physical and military‐specific training loads.

Results here support previous military training studies, whereby an inverse relationship exists between initial fitness (cardiorespiratory and strength/power) and subsequent performance changes; such that the slower/weaker recruits at entry improved the most, while those presenting with higher fitness levels displayed no change or decreased performances.^5^, ^6^, ^8^, ^11^, ^14^, ^18^, ^23^, ^35^ Despite this well‐established trend, this association has rarely been directly examined in BT, particularly using appropriate statistical analysis. Importantly, for recruits of high fitness, these data suggest that the physical demands and progression experienced during BT may not be sufficient to elicit positive training responses, or they are already well trained that they have less capacity for improvement. Of relevance, having a high initial fitness level is an important component for successful task performance during military service that functions to reduce the incidence of injuries or discharge.^23^ Unlike many individual athletic pursuits, military training tends to be completed in groups with a range of different physical fitness attributes, with an end‐goal of getting all members to meet minimum PES. To avoid detraining of the fittest recruits, streamed PT (i.e., individual training groups of recruits split by alike fitness) may provide greater exercise stimulus and subsequent physical adaptations for these recruits,^8^ as fully individualized training programmes are not practically viable. Conversely, the significant improvements occurring in entrants of lower fitness in the present study highlights the efficacy of BT programming in developing recruit strength and cardiorespiratory fitness. These findings indicate that BT increased the fitness of the least fit recruits,^14^ which will benefit performance on military‐specific tasks that are common to all within a particular role, such as load carriage or team lifting and carrying tasks.^2^

Regarding gender, the review by Varley‐Campbell et al.^36^ comprising 29 military training studies concluded that overall physical performance improves, and by a similar margin between men and women, over the course of BT. However, clear physical performance differences are apparent between genders at entry that are not negated throughout training (i.e., men typically perform better pre‐ and remain higher post‐BT, when compared with women).^36^ Similarly, we observed, on average, that women started training with lower levels of physical performance (aerobic endurance and muscular strength/power) and, in unadjusted absolute terms, made predominantly similar relative improvements to men. However, when estimating changes across the spectrum of baseline physical performance scores, improvements in women were lower (or decrements higher) in all tests and courses. Similarly, this is a combination of higher average fitness in men, and when higher average improvement is evident in women, so too is a stronger association between entry fitness and performance change. Importantly though, although separate slopes to determine estimated marginal means to reduce between‐group bias were used, the non‐random imbalance in fitness and range of values between men and women at baseline means these estimates may contain unmeasured biases and some extrapolate outside the female cohort data. Given the different gradients observed across tests and courses when applying the interaction model, it is not possible to generalize on the overall effect of entry fitness on apparent imbalances in fitness improvement between men and women. It may be expected that men and women adapt differently to training considering the combination of sex differences in physiology and performance,^37^ as well as the differing physical demands experienced during BT.^4^ When graphically matched for entry performance, men made more considerable improvements relative to women in all tests. A comparable result was reported recently by Santtila et al., with men showing greater training adaptations in all fitness variables compared with women of the same fitness over 12 months of military service, suggesting that a fitness gap between men and women continues, and may even be exacerbated further, long‐term, and in some components of fitness more than others.^23^ Consequently, accounting for physical performance at the start of training identifies the training needs of individuals thus highlighting the need for tailored training programmes to enhance the capability of recruits as they progress into specialized training and service.

The tests performed at the start and end of BT form part of the British Army's PES, which are empirically linked to essential occupational task performance and assess targeted components of fitness (cardiorespiratory fitness and upper‐ and lower‐body muscular strength/power). Therefore, the changes we observed over training and the differences between courses and men and women may also relate to the type of physical capacity being trained and assessed, particularly in large recruit cohorts.

Overall, cardiorespiratory fitness adaptations were observed following SE and JE BT programmes, the greatest 2 km performance improvements were observed in JE‐Long recruits (unadjusted mean: 7.7%), compared to SE (6.8%) and JE‐Short (4.6%). The pre‐post improvement seen in SE is slightly lower compared to previous British Army SE cohorts' 2.4 km run performance (9.1% and 10.0%).^4^, ^6^ This is, potentially a result of the larger sample used in the present study and reflective of the changes in the PT syllabus (inclusion of more functional strength and conditioning training, compared to the previous training programmes [as detailed in Williams et al.^7^] that were highly focused on cardiorespiratory training). Notably, while JE‐Long recruits entered training at a similar level of fitness to SE, the longer duration of this course, and maturation of the recruits (who are younger on entry), potentially afforded greater training adaptations to occur in those of lower baseline fitness (largest impact of entry fitness on subsequent change). Only one study has assessed changes in cardiorespiratory fitness over British Army JE courses, which reported a 2.4% reduction in predicted maximal oxygen uptake (V̇O_2max_) following JE‐Short, and a modest increase following JE‐Long (3.0%) in male infantry recruits.^5^ These results were attributed to high initial fitness (V̇O_2max_ ~ 58.0 mL∙kg^−1^∙min^−1^) of these cohorts and the emphasis on lower intensity endurance training during BT programmes at this time.

The overall level of improvement in 2 km run performance observed between men and women in the present study was similar. This finding is contrary to the review by Varley‐Campbell et al.^36^ where, when looking specifically at cardiorespiratory fitness, women experienced greater pre‐post training improvement than men (10.4% vs. 5.7%, respectively) across 12 studies (run times assessed over 1.6–3.2 km). Moreover, here, when graphically matching for entry run performance, the response to training was greater in men across all courses. Because all recruits complete the same training programmes, it is common in military training that when entry fitness is not matched, individuals work at different intensities, producing potentially different adaptive responses. For example, when intensity was not matched in BT, women experienced greater levels of cardiovascular strain, attributed to lower initial fitness, and saw greater improvements compared to men (6.8% vs. 4.1%).^4^ When men and women have been trained separately to match relative intensities, Richmond et al.^6^ reported the same improvement for men and women. These findings suggest that the level of improvement between genders is not only attributed to entry fitness, but also the training intensity, which supports the notion to segregate training by gender to better manage training load.^37^

In past decades, physical fitness assessments in military training have focused on muscular endurance of the upper body and trunk (usually via pull‐ups, push‐ups, and sit‐ups). With greater understanding of components of fitness and factors commensurate with essential military tasks, the focus of both training and testing has more recently shifted to assessments of muscular strength and power in various military organizations.^16^ The British Army now assess lower body muscular strength and upper body power using the MTP and MBT, respectively. A regression to the mean in MTP and MBT was clear, with loss of performance in high‐fit entrants and gain in performance in low‐fit entrants. On average, we saw the most substantial improvements in JE‐Long (overall and baseline‐adjusted) for both strength tests compared to the other courses, which may be a combined product of course length and maturation. In comparison to run performance, the two strength‐based tests showed more variability and divergence of responses. The comparison between men and women is also limited by both overall sample size of women lacking in the JE courses and of women with the highest MBT and MTP performance. Focusing on SE, the steep training slopes for these tests in both men and women suggest that the 14 weeks of training elicit considerable gains in lower body strength (versus aerobic and upper body strength), which is likely a result of the (potentially unaccustomed) combined strength training tasks undertaken during BT (i.e., strength and conditioning, and load carriage). This, and the greater average improvements in women, is consistent with Varley‐Campbell et al.,^36^ who determined greater median pre‐post improvements in women compared to men for both upper (W: 13.0% vs. M: 6.9%) and lower (W: 10.5% vs. M: 7.0%) body strength assessed via tests such as bench/leg press.

When looking at individual responses, courses longer than SE show that the effect of physical performance at the start of training affords greater performance improvements for men than women in upper (MBT in JE‐Long) and lower (MTP in JE‐Short and JE‐Long) body strength, irrespective of the women's lower physical performance at the start of training. Albeit, while women still benefited from training, the lack of improvement over the longer courses (compared to men) suggest that strength training is not providing adequate long‐term stimulus after initial adaptation. Indeed, it has been suggested that a training plateau may be achieved due to most neuromuscular adaptations occurring during the first 8 weeks of BT, with no further gains observed during an additional 8 weeks of training.^12^ Brock & Legg^38^ reported that women had significant gains in strength (maximal isometric upright pull and maximal incremental dynamic lift) following only 6 weeks of BT, highlighting that women respond quickly to training and therefore may require increases in training variability/stimulus over longer training periods to promote lasting adaptations. Further, it is also possible that the resultant strength gains may be overcome by aerobic training improvements as concurrent strength and endurance training has shown to inhibit explosive strength development when compared to strength training alone.^39^ In addition to this, performing high levels of low‐intensity endurance activity resultant of military training (e.g., combat training, marching/drill, range shooting) could negate the effect on strength and aerobic outcomes from PT if programming and recovery between activities is not periodised appropriately.^40^ Conversely, the longer training durations of JE courses in the present study afford men significant upper and lower body strength gains relative to SE, and to women. In the study by Legg & Duggan,^5^ all indices of isometric muscular strength in men improved to a greater extent during JE‐Long (vs. JE‐Short and SE) indicating that lengthier durations of PT promote concomitant strength improvements. Data gathered in other British Army cohorts have also shown that there is a low overlap (high performing women outperform low performing men) in the Powerbag lift strength test.^36^ Taken together with data in the present study, these findings indicate that strength may be the fitness component requiring greatest attention for women when translated to military tasks; particularly since manual handling and load carriage are critical tasks requiring high levels of functional muscle strength.^8^ Thus, the addition of the MBT and MTP (and updated strength and conditioning programming provided in BT) that are linked more closely to the physical actions performed during essential military tasks should promote more task‐relevant strength outcomes as recruits progress through training.^41^

While not directly measured, the performance changes over different BT courses could also be attributed to the age of recruits entering training, and their subsequent maturation and fitness development occurring in response to a new stimulus. Specifically, JE recruits enter BT between the ages of 16–17.5 years, which is seen as a crucial physical maturation period for accelerated neuromuscular adaptations and trainability.^42^ Further, upon the onset of the adolescent growth spurt, maturational differences are apparent between genders for nearly all components of fitness.^43^ Specifically, men see a greater magnitude of growth spurt resulting in marked acceleration in strength throughout the late teens/early twenties (peak age for hypertrophy), while women's strength increases linearly with age due to an earlier growth spurt, and therefore, few women outperform men after the age of 16.^44^, ^45^ This growth, and the associated increases in muscle mass, is particularly reflected in the greater slopes of improvement in men's strength, compared to women, following JE‐Long and in the analysis where recruits were grouped based on physical performance at the start of training. Consequently, for women, more variation in strength training stimulus (type/intensity/volume) and periodisation could be applied over longer training courses to promote continuous development (and avoid plateau), including deconfliction with military‐specific training activities. Crucially, it has been suggested that functional strength training should be focused on during this period as it can lead to improvements in load carriage alone,^2^ while aerobic conditioning should not be the main focus until adulthood (18+) where it appears to be very trainable.^43^ This is reflected in the training syllabus (Table 1) whereby JE dedicates a significant proportion of strength development to gym‐based sessions, as opposed to SE that perform abundant load carriage exercise. On the other hand, the average age of SE recruits is typically older as recruits can enter BT from the age of 17.5 up to 35 years. Therefore, SE recruits are likely to commence training at a higher level of physical maturity with prior experience in PT, compared to JE. Importantly, further improvements in strength training‐induced hypertrophy are much more limited in trained than in untrained individuals,^45^ which may explain the larger performance improvements seen among the JE cohorts on all three tests, compared to SE. Therefore, although JE recruits are entering training at a younger age and likely lower levels of physical maturity than in SE, this indicates that the effects of maturation (peak strength and hypertrophy age), combined with longer adaptation time, could lead to a greater level of improvement in physical capabilities. Of relevance, the overall entry performance for all three tests were also higher in SE than JE courses, justifying the importance of programming and assessing adaptations to training based on physical performance at the start of BT. In the future, it would therefore be of interest to determine how well fitness is maintained as recruits progress out of BT, and whether the fitness differences post‐BT (between course and gender) affords any advantage during subsequent training and service (i.e., performance of RFT(S)).

The present study is the largest to quantify changes in physical performance during British Army BT to date, and the first to describe course and gender training differences in relation to the recently introduced tests; however, there are still some limitations to note. Due to data only covering those individuals who completed the tests pre‐ and post‐BT (survival bias); we were unable to determine the first‐time pass rates and attrition rates (due to injury, voluntary discharge, back‐trooping etc.), which could emphasize any differences in physical demands of the courses on capability between gender. Further, no data were obtained regarding injury rates during training, which again may highlight patterns in response to training between genders. As described in the statistical approach, this study was of observational design, and therefore using a traditional ANCOVA model to “adjust” for entry fitness would have been biased.^20^, ^21^ Yet, we still caution that the estimates and estimated marginal means produced could be influenced by the larger sample size, higher mean fitness and spread of scores in men, and partly, or wholly, by unmeasured and uncontrolled biases of an observational human field trial. However, as a descriptive analysis, we believe using this association to present estimated change on a range of entry scores is an improvement on previous analyses of military training data without introducing much more complicated models to account for individual variation in responses and imbalances of group size between gender and courses. Nevertheless, participants are representative of the wider population and form a large proportion of recruits who undertake SE and JE BT programmes in the British Army (≈7000 per year). Regarding women specifically, while the difficulty in drawing comparisons to men due to imbalanced sample sizes remains, the representation in the data was SE: 11.4%, JE‐Long: 7.1%, JE‐Short: 16.8%. These figures are representative of the wider Army at the time of the research (female intake in 2020: 10.9%^24^) which is particularly important given women are typically underrepresented in research.^46^

Overall, our study showed physical fitness improvement (cardiorespiratory endurance and muscular strength/power) during standardized BT across 14 (SE), 23 (JE‐Short) and 49 (JE‐Long) weeks, with wide inter‐individual variation. In all tests and courses, we observed a form of “regression to the mean” whereby those with lower fitness at entry made greater improvements than those possessing high entry fitness. Examining the relationship with physical performance at the start of BT, rather than solely change scores revealed differences between courses and men and women in physical fitness improvement, indicating a large proportion of improvement observed in women could be attributed to their lower fitness at entry. Future analyses and training should assess this relationship and either account for physical performance at the start of BT or present training‐related change across a distribution of starting fitness scores, to better understand how individuals of different fitness levels adapt to training, as well as the differing training responses between men and women. As BT is a crucial period for development in physical fitness, consideration of these factors may be beneficial for future success of military job‐task performance.

## 5 PERSPECTIVE

British Army BT (both SE and JE) improves cardiorespiratory fitness and muscular strength in many, but not all recruits. Changes in fitness depend on the course and gender, and, in large part, on individual fitness levels at the start of BT. Recruits entering BT at high fitness levels should be afforded the opportunity to improve (and mitigate decline) through streamed training programmes. To better understand how individuals of different fitness levels, and gender, adapt to training, future analyses should present training‐related change across the distribution of starting fitness scores, and practitioners should account for the relationships in physical performance differences to inform training prescription. Women, particularly JE recruits, may need more targeted and progressive strength training programmes to avoid training plateau over the course duration. Single sex training may therefore better allow for intensity‐based programming, including separation based on physical performance at the start of training. However, it is recognized this may not always be practicable when training multiple large cohorts. Nevertheless, consideration of these factors in BT are important for enhancing future training for optimal performance of military tasks.

## Data Availability

Research data are not shared.
